# Determinants of mobility in community-dwelling older adults: a network analysis before, during, and two years after the onset of the COVID-19 pandemic

**DOI:** 10.1093/gerona/glaf162

**Published:** 2025-07-22

**Authors:** Katja Lindeman, Taina Rantanen, Essi-Mari Tuomola, Johanna Eronen, Laura Karavirta, Kaisa Koivunen

**Affiliations:** Faculty of Sport and Health Sciences, Gerontology Research Center, University of Jyväskylä, Jyväskylä, Finland; Faculty of Sport and Health Sciences, Gerontology Research Center, University of Jyväskylä, Jyväskylä, Finland; Faculty of Sport and Health Sciences, Gerontology Research Center, University of Jyväskylä, Jyväskylä, Finland; Faculty of Sport and Health Sciences, Gerontology Research Center, University of Jyväskylä, Jyväskylä, Finland; Faculty of Sport and Health Sciences, Gerontology Research Center, University of Jyväskylä, Jyväskylä, Finland; Faculty of Sport and Health Sciences, Gerontology Research Center, University of Jyväskylä, Jyväskylä, Finland

**Keywords:** complex systems, life-space mobility, autonomy

## Abstract

**Background:**

We conceptualized out-of-home mobility as life space mobility and autonomy outdoors. Both are correlated with quality of life and influenced by multiple underlying factors. We used a complex systems approach and network models to explore changes in networks consisting of out-of-home mobility indicators and their determinants before, during and two years after the onset of the COVID-19 pandemic.

**Methods:**

Participants were older adults aged 75, 80 and 85 years at baseline (2017–2018), with follow-ups in 2020 and 2021–2022 (*n* = 607). Life-space mobility, autonomy outdoors, and socio-demographic, physical, psychosocial, financial, and environmental determinants were assessed using the same validated scales at all three time points. Mixed graphical model networks were estimated for each time point. Differences in network properties and the relative importance of determinants associated with life-space mobility and autonomy outdoors were compared across time points.

**Results:**

During the COVID-19 pandemic, both life-space mobility and autonomy outdoors declined (*P* < .05). Throughout the follow-up, walking difficulty and sex remained consistently associated with life-space mobility and psychosocial factors with autonomy outdoors. At the onset of the pandemic, being female (vs male) was more strongly associated with reduced autonomy outdoors than at other times, while the associations with older age and poorer health were weaker.

**Conclusions:**

The pandemic reduced older adults’ out-of-home mobility and altered the factors underlying it. During the pandemic, environmental support for out-of-home mobility diminished as destinations of interest for older people were closed. This especially affected women, potentially leading to less favorable participation trajectories in the future.

## Introduction

Out-of-home mobility is a key aspect associated with healthy aging, enabling older adults to engage in activities they value and access community amenities.^1^^,^^2^ We conceptualized out-of-home mobility as consisting of life-space mobility and autonomy outdoors, with the ability to move as their underlying factor. Life-space mobility refers to spatial movement in one’s living environment and is defined as the extent of movement, either independently or with the help of assistive devices or transportation, within an environment that extends from one’s home to the neighborhood and beyond.^3^ Autonomy in outdoor mobility encompasses an individual’s sense of control and satisfaction over decisions about where, when, and how to move.^4^^,^^5^ Higher perceived autonomy outdoors often coexists with higher life-space mobility.^6^ Nevertheless, they can be considered two distinct yet related dimensions of out-of-home mobility^7^: while life-space mobility captures the extent of actual movement within one’s everyday environment, autonomy outdoors reflects perceived satisfaction with one’s opportunities to access desired destinations and participate in activities outside the home.

According to Webber et al.’s conceptual framework, the out-of-home mobility of older adults is influenced by physical, psychosocial, cognitive, financial, and environmental determinants.^3^ Additionally, socio-demographic factors such as sex, life history, and culture have an overarching influence on the mobility of older adults.^3^ Empirical studies have suggested that broadly the same determinants underlie both life-space mobility and autonomy outdoors. Physical determinants, such as lower extremity performance, may influence life-space mobility more.^6^^,^^8-10^ Meanwhile, those who perceive fewer environmental barriers and continue to pursue their goals, adapting them to better match their current resources, may perceive greater autonomy outdoors despite functional decline.^5^^,^^11^

However, out-of-home mobility and the relative importance of its underlying determinants may vary in different contexts.^3^ Previous studies have shown that the COVID-19 pandemic has affected both older adults’ life-space mobility and their perceived autonomy outdoors.^12^^,^^13^ Efforts to restrict the spread of the virus during the pandemic reduced environmental support for outdoor mobility by diminishing the incentives to leave the home when desired community destinations were closed, social activities were suspended, and external social support decreased. To date, studies investigating the impact of the COVID-19 pandemic on the mobility of older adults have mainly focused on physical determinants, with less attention paid to other determinants such as psychosocial, cognitive, financial, and environmental aspects.^14^ It is also still unclear what role different mobility determinants have played and whether their relative importance for out-of-home mobility—specifically, life-space mobility and perceived autonomy outdoors—has changed during the pandemic. Conversely, simultaneously examining the association of multiple determinants with out-of-home mobility indicators is challenging, particularly when using traditional statistical methods, which typically isolate specific associations and control for potential confounding factors.

The complex systems approach, which seeks to understand how interrelated elements form a whole, has been increasingly applied to address complex issues related to public health. Statistical network models, which describe multivariate dependency structures, are one application of this approach.^15^ These network models have become particularly popular in psychology^16^ and are also being adopted in other disciplines. Recently this perspective has been proposed to study functional aging^17^ and has been adopted, for example, to better understand the complexity of frailty in aging^18^^,^^19^ and to explore the interplay between determinants that shape the intrinsic capacity of older adults.^20^ In this study, we aimed to apply the complex systems approach and network models to explore the interrelationships between determinants affecting older adults’ out-of-home mobility, as outlined in the Webber et al. conceptual framework.^3^ Additionally, we aimed to examine the temporal nature of these determinants during the COVID-19 pandemic and to determine whether the relative importance of certain determinants related to life-space mobility or perceived autonomy outdoors changed during this period.

## Methods

### Study design and setting

The data for this study form part of the cohort study “Active aging—resilience and external support as modifiers of the disablement outcome” (AGNES). AGNES is a population-based cohort study with three age groups (75-, 80-, and 85-year old at baseline in 2017–2018) of people living independently in the city of Jyväskylä.^21^ Data for this study were gathered at three time points: a structured home interview in 2017–2018 (T1, *n* = 1021), a postal questionnaire in May and June 2020 at the onset of the COVID-19 pandemic (T2), and a structured home interview in 2021–2022, when the COVID-19 pandemic had been ongoing in Finland for about two years (T3). Details of the baseline protocol and recruitment process,^21^^,^^22^ as well as participation in the COVID-19 survey in 2020^23^ and follow-up measurements in 2021–2022,^13^ have been previously documented elsewhere.

### The COVID-19 pandemic in Finland during the follow-up studies

The COVID-19 postal questionnaire in 2020 took place during the enforcement of the Emergency Powers Act in Finland (March 16—June 16, 2020). During this period, all social, cultural, and community activities were suspended, and public gatherings were limited to ten people. People over 70 were advised to be extra cautious and to remain in quarantine-like conditions until June 22, 2020.^24^^,^^25^ Studies show that during the first months of the pandemic, three-quarters of older adults reduced social engagement,^26^ with almost 90% of people over 70 reporting fewer physical contacts.^27^ During the second follow-up in 2021–2022, restrictions were more regionally targeted and adapted to infection rates, following the government’s hybrid strategy. In February 2022, the government revised its hybrid strategy to avoid large-scale restrictions, focusing on voluntary health-conscious behavior, and vaccination to reduce the severe consequences of infection.^28^

### Variables included in this study

Data on the variables used were collected at all three time-points. Indicators of out-of-home mobility were life-space mobility and perceived autonomy outdoors*. Life-space mobility* was assessed using the University of Alabama at Birmingham Study of Aging Life-Space Assessment (LSA) questionnaire, which assesses the extent of a person’s life-space mobility, the frequency of movement within the area, and the need for assistance over the past four weeks. The composite score ranges from 0 to 120 points, with higher scores indicating greater life-space mobility. The validity and test-retest reliability of the LSA in the older population have been established in previous studies.^22^^,^^29^  *Autonomy outdoors* was assessed using the “autonomy outdoors” subscale of the Impact on Participation and Autonomy (IPA), which assesses an individual’s perceived satisfaction with their opportunities to access desired destinations and participate in activities outside the home. These activities include visiting family and friends, traveling and going on trips, spending leisure time, meeting other people, and living according to personal preferences. Each item is rated on a scale from zero (very good) to four (very poor). The total score ranges from 0 to 20, with higher scores reflecting reduced autonomy. Previous studies have demonstrated the validity and test-retest reliability of the IPA, as well as its validity for older adults.^30–32^

Physical determinants included self-reported difficulty in walking 2 km and self-reported health status. *Difficulty in walking 2 km* was assessed with the question: “Can you walk about 2 km?” with five response options, from (I) can do it without difficulty to (V) cannot do it even with help. Self-­reported walking difficulty has been shown to be a valid measure of early signs of disability in older adults.^33^ Self-reported *health status* was assessed as follows: “How would you rate your current health status in general?” with the five response options, from (I) very good to (V) very poor. Self-rated health has been shown to be a feasible measure of overall health and to have predictive value, for example, for mortality in older adults.^34^ Psychosocial determinants included self-reported marital status, loneliness, and depressive symptoms. *Marital status* was assessed by the question “What is your current marital status?” with the five response options: never married; married; cohabiting; widowed; and divorced. *Loneliness* was asked as follows: “How often do you feel lonely?” with the four response options, from (I) very rarely/never to (IV) almost always. *Depressive symptoms* were assessed using the 10-item Center for Epidemiologic Studies Depression Scale (CES-D-10) questionnaire, where the person is asked to rate the frequency of each depressive symptom (eg, “I felt depressed”) in the past week on a four-point scale: 0 rarely or never—3 almost always. The total score ranges from 0 to 30 points, with higher scores indicating more perceived depressive symptoms.^35^ The CES-D-10 has previously been shown to be valid for identifying depressive symptoms in older adults.^36^ Environmental determinants included the perceived safety of the neighborhood and the population density within 1 km of the participant’s home. Information on the *perceived fear of moving around the neighborhood* was obtained by asking the question: “Are you afraid of anything when you move around your neighborhood?” with the response options: no; yes. *Population density* (number of inhabitants per 1 km^2^) describes the availability of services and the amount of infrastructure enabling outdoor movement. Its calculation was based on 1 × 1 km^2^ population grid data. Participants’ addresses were geocoded using Digiroad datasets from different points in time,^37^ and the population numbers of the 1 × 1 km^2^ grid datasets were also from different points in time^38^ to match the time points of the study. The population density within 1 km of the participant’s home was calculated at each time point. If a person moved between time points, the population density was calculated based on the new address. Economic determinant included self-reported *financial situation,* which was assessed with the question: “How would you rate your financial situation?” with the five response options, from (I) very good to (V) do not know.

Additionally, we included sex, age, and total years of education to represent cross-cutting socio-demographic determinants. Sex and age were obtained from the Digital and Population Data Services Agency, and total years of education were self-reported at baseline. Due to the restrictive measures during the COVID-19 pandemic, we were unable to assess participants’ cognitive function in 2020. To ensure comparability across all time points, the cognitive determinant was excluded from the analysis, although it is a component of the framework presented by Webber et al.^3^

### Statistical analyses

#### Data preprocessing and descriptive analysis

A total of 607 people who participated at all three time points made up the analytical sample for this study. There was some missing data across variables, with the highest proportion being 6.6%. Missing data were handled using available variable information with multiple imputation procedures in IBM SPSS Statistics for Windows, version 28.0 (IBM corp., Armonk, NY).

To facilitate the interpretation of the direction of associations between variables in the network models, and due to the limited responses at the extremes of many categorical variables, categorical variables were converted to binary form. Difficulty in walking 2 km was categorized as *“no difficulty in walking”* (response option I: can do it without difficulty) and *“at least some difficulty in walking”* (response options II–IV: can do it but with some difficulty; can do it but with a lot of difficulty; cannot do it without help from another person; cannot do it even with help). Self-reported health status was categorized as *“good health”* (response options I–II: very good; good) and *“fair or poor health”* (response options III–V: fair; poor; very poor). Marital status was categorized as *“partnered”* (response options: married; cohabiting) and *“not partnered”* (response options: never married; widowed; divorced). Loneliness was categorized as *“never or very rarely”* (response option I: very rarely/never) and *“at least sometimes”* (response options II–IV: rarely; often; almost always), and financial situation was categorized as *“good”* (response options I–II: very good; good) and *“fair or poor”* (response options III–V: fair; poor; do not know). Coding and the reference categories for binary variables are shown in Supplementary Table S1. As the scales for life-space mobility and autonomy outdoors are contradictory (a higher score indicates greater life-space mobility but lower autonomy outdoors), the subscale for autonomy outdoors was inverted, meaning that in this study a higher score indicates better autonomy outdoors, which facilitates the interpretation of the networks.

Descriptive data are presented as means and standard deviations (SDs) for continuous variables, and as proportions and frequencies for binary variables. Differences between time points were assessed using Cochran’s *Q* test for binary variables and repeated measures ANOVA with Bonferroni correction for continuous variables. Additionally, repeated measures ANOVA with Bonferroni correction was used to analyze changes in life-space mobility and autonomy outdoors over time, both for all participants and stratified by sex, and to assess time × sex interactions, both overall and separately between T1 and T2, and between T2 and T3. Analyses were conducted using IBM SPSS Statistics for Windows, version 28.0.

#### Network estimation and visualization

Network models consist of nodes representing observed variables and edges representing statistical relationships between nodes. A mixed graphical model (MGM), which can handle mixed types of variables (continuous, categorical, binary),^39^ was used to analyze the networks between indicators of out-of-home mobility—life-space mobility and autonomy outdoors—and the potentially influencing physical, psychosocial, environmental, financial, and socio-demographic determinants. Networks were estimated separately for three different time points (T1, T2, and T3).

For the network analyses, the R package *mgm*^39^ was used to estimate the pairwise weighted adjacency matrix between the variables. The *binarySign* command was employed to determine the sign of interactions within binary nodes and between binary and continuous nodes. All relationships in the networks represent pairwise interactions (*k* = 2), with each estimated relationship between two variables accounting for the influence of all other variables in the network. Consequently, the absence of a relationship between two variables implies that they are conditionally independent, given the presence of all other variables. The Least Absolute Shrinkage and Selection Operator (LASSO) was applied to generate sparse networks by shrinking smaller coefficients toward zero, thereby minimizing false positive associations between nodes to achieve a more conservative network estimate. Additionally, the Extended Bayesian Information Criterion (EBIC), which balances model fit and complexity, was used with its hyperparameter set at 0.50, as previously recommended.^40^ Nodewise predictability, that is, how well a node is predicted by all other nodes connected to it in the network,^41^ was calculated for life-space mobility and autonomy outdoors and indicated by the proportion of variance explained (R2).

The *qgraph* package^42^ was used to visualize the edge-weight matrix as a network based on Fruchterman and Reingold’s algorithm, which generates plots with the most strongly connected nodes placed in the center of the graph and the weakly connected nodes at the periphery. However, to ensure visual comparability between the networks, the average layout of the three individual layouts from different time points was applied when plotting the network models. As we only had continuous and binary variables, green edges in the network models indicate positive associations, red edges indicate negative associations, and edge thickness represents the strength of the conditional dependence.

Standardized absolute pairwise edge weights, which represent the strength of conditional dependencies, were examined to assess the relative importance of different determinants on life-space mobility and autonomy outdoors. As we only had continuous and binary variables, the direction and relative importance of all variables could be derived from the weighted adjacency matrix. Heat maps were used to visualize the ranking of the standardized absolute edge weights associated with the nodes of life-space mobility and autonomy outdoors, and to assess the temporal changes in the relative importance of determinants across waves (T1–T3).

The network structures (T1 vs T2 and T2 vs T3) were compared using the three tests included in the Network Comparison Test (NCT): global strength (ie, whether the overall connectivity differs between two networks), structural invariance (ie, whether the pattern of connections differs between networks), and if a structural variance was detected, differences in individual edges were tested using the Benjamini–Hochberg correction (ie, which specific edges are different between the networks).^43^ As the same participants were assessed three times, the paired version of the NCT was used.

Post hoc stability analyses were conducted to assess the accuracy of the network estimation in terms of edge weight parameters. To assess the stability of the edge weight estimates, 95% confidence intervals (95% CI) were calculated using the nonparametric bootstrap method. A wider 95% CI indicates greater instability in the estimates. Note that the plots indicate the stability of the edge estimates, but do not indicate whether the edges are significantly different from zero. The bootstrapped difference test (significance level *P* < .05) was used to determine whether there were statistically significant differences between edges for each pair of non-zero nodes in the estimated network. For the bootstrapped analyses, the dataset was resampled 2000 times using the R package *bootnet.*^44^ All network estimations and graphical visualizations were conducted in R version 4.4.1 (R Core Team, 2024) (R Foundation for Statistical Computing, Vienna, Austria).

## Results

Descriptive characteristics of the participants are shown in Table 1. At baseline in 2017–2018 (T1), 58.0% of the participants were women. The mean age of the participants was 78.3 (SD 3.3) years, and the mean total years of education was 11.9 (SD 4.3) years. At follow-up in 2021–2022 (T3), the mean age was 82.2 (SD 3.3) years, and the mean follow-up time was 3.9 (SD 0.3) years. From T1 to T2, statistically significant negative changes were observed in depressive symptoms and in the proportion of participants experiencing walking difficulty, living alone, feeling lonely, and being afraid of moving in the neighborhood. From T2 to T3, a statistically significant recovery was observed in depressive symptoms, but the proportion of participants experiencing walking difficulty, poorer self-reported health, living alone, and feeling lonely increased (Table 1).

**Table 1.** Descriptive characteristics of the participants (*n* = 607).

	Baseline 2017–2018 T1	May–June 2020 T2	Follow-up 2021–2022 T3	*P*
**Sociodemographics**				
Women, % (f)	58.0 (352)	-	-	
Total years of education, mean (SD)	11.9 (4.3)	-	-	
Age in years, mean (SD)	78.3 (3.3)	80.3 (3.2)	82.2 (3.3)	<.001^a^ ^b^ ^c^
**Physical determinants**				
No difficulty walking 2 km, % (f)	69.2% (420)	64.9% (394)	61.9% (376)	<.001^a^ ^c^
Good self-rated health, % (f)	54.9% (333)	52.9% (321)	42.8% (260)	<.001^b^ ^c^
**Psychosocial determinants**				
Living with a partner, % (f)	60.5% (367)	57.5% (349)	55.7% (338)	<.001^a^ ^c^
Feeling loneliness never or very rarely, % (f)	59.8% (363)	44.0% (267)	42.7% (259)	<.001^a^ ^c^
Depressive symptoms (CES-D-10, score 0–30), mean (SD)	4.8 (4.1)	7.7 (4.6)	6.4 (4.3)	<.001^a^ ^b^ ^c^
**Environmental determinants**				
No fear of moving in the neighborhood, % (f)	96.7% (587)	92.1% (559)	94.6% (574)	<.001^a^
Population density (inhabitants/1 km²), mean (SD)	1868.2 (1283.5)	1941.5 (1370.6)	2006.9 (1414.4)	<.001^a^ ^b^ ^c^
**Economic determinants**				
Good self-reported financial situation, % (f)	62.8% (381)	66.7% (405)	67.9% (412)	.020^c^

Statistical differences between time points were assessed using repeated measures ANOVA with Bonferroni correction for continuous variables, and Cochran’s *Q* test for binary variables.

Abbreviations: CES-D-10 = the 10-item Center for Epidemiologic Studies Depression Scale; f = frequencies; SD = standard deviation.

a T1 vs T2.

b T2 vs T3.

c T1 vs T3.

Life-space mobility and autonomy outdoors, along with their changes at different time points for all participants and according to sex, are presented in Table 2. At baseline, participants had an average life-space mobility score of 74.5 (SD 17.8) points and an average autonomy outdoors score of 15.2 (SD 3.6) points. Life-space mobility decreased by an average of 9.5 (SD 20.8) points from T1 to T2 (*P* < .001) and increased by an average of 2.1 (SD 21.2) points from T2 to T3 (*P* = .050). Autonomy outdoors decreased by an average of 6.9 (SD 5.4) points from T1 to T2 (*P* < .001) and increased by an average of 5.0 (SD 5.5) points from T2 to T3 (*P* < .001). At all time-points, women showed lower levels of life-space mobility and autonomy outdoors. At the onset of the COVID-19 pandemic, they also experienced a greater decline in life-space mobility (men: –6.5 [SD 22.6] vs women: –11.7 [SD 19.1]; time × sex interaction between T1 and T2: *P* = .002) and autonomy outdoors (men: –5.9 [SD 5.2] vs women: –7.6 [SD 5.4]; time × sex interaction between T1 and T2: *P* < .001). From T2 to T3, a statistically significant recovery in life-space mobility was observed only among women. Autonomy outdoors improved statistically significantly for both men and women, with no statistically significant time × sex interaction.

**Table 2.** Life-space mobility and autonomy outdoors and their changes at different time points for all participants and by sex.

	**T1 2017**–**2018**	**Change T1**–**T2**	T2 2020	**Change T2**–**T3**	**T3 2021**–**2022**	Time effect		Time × sex interaction overall		Time × sex interaction T1 vs T2		Time × sex interaction T2 vs T3	
	Mean (SD)	Mean (SD)	Mean (SD)	Mean (SD)	Mean (SD)	*F*	*P*	*F*	*P*	*F*	*P*	*F*	*P*
**Life-space mobility (score 0**–**120)**													
All participants (*n* = 607)	74.5 (17.8)	−9.5 (20.8)	65.0 (23.4)	+ 2.1 (21.2)	67.1 (10.2)	74.6	<.001^a^ ^c^	5.4	.005	9.4	.002	5.1	.025
Men (*n* = 255)	80.5 (17.2)	−6.5 (22.6)	74.1 (22.5)	+0.2 (21.7)	73.9 (19.3)	16.9	<.001^a^ ^c^						
Women (*n* = 352)	70.1 (17.0)	−11.7 (19.1)	58.4 (21.8)	+3.7 (20.7)	62.1 (19.5)	66.1	<.001^a^ ^b^ ^c^						
**Autonomy outdoors (reversed score 0**–**20)**													
All participants (*n* = 607)	15.2 (3.6)	−6.9 (5.4)	8.3 (5.2)	+5.0 (5.5)	13.4 (4.2)	633.1	<.001^a^ ^b^ ^c^	9.0	<.001	15.4	<.001	3.3	.068
Men (*n* = 255)	15.8 (3.1)	−5.9 (5.2)	9.9 (5.0)	+4.5 (5.2)	14.4 (3.6)	221.2	<.001^a^ ^b^ ^c^						
Women (*n* = 352)	14.8 (3.9)	−7.6 (5.4)	7.2 (5.1)	+5.4 (5.7)	12.6 (4.5)	422.1	<.001^a^ ^b^ ^c^						

The subscale of autonomy outdoors is inverted, that is, higher scores indicate greater autonomy. Statistical difference between time points and sex × time interaction assessed using repeated measures ANOVA with Bonferroni correction.

Abbreviations: Autonomy outdoors = autonomy outdoors subscale of the Impact on Participation and Autonomy; SD = standard deviation; life-space mobility = the University of Alabama at Birmingham Study of Aging Life-Space Assessment.

a T1 vs T2.

b T2 vs T3.

c T1 vs T3.

**Figure 1.**
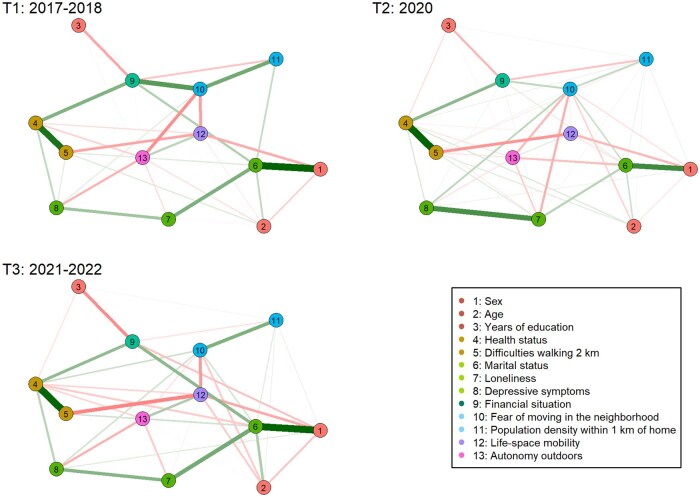
Networks of mobility determinants associated with life-space mobility and autonomy outdoors across T1-T3. Edge thickness indicates the magnitude of the associations between nodes. For ease of interpretation, the subscale of autonomy outdoors is inverted, that is, higher scores indicate greater autonomy.

A consistent set of physical, psychosocial, environmental, financial, and socio-demographic determinants potentially influencing life-space mobility and autonomy outdoors were included to estimate the network in each period, optimizing the comparability of the networks across T1–T3 (Figure 1). Overall, the directly connected nodes explained 32.9%, 30.0%, and 39.2% of the variance in life-space mobility and 34.7%, 25.1%, and 38.6% of the variance in autonomy outdoors in T1, T2, and T3, respectively.

In T1, the number of nonzero edges were 38 (density 0.49, ie, 49% of all possible connections); in T2, the number of nonzero edges were 50 (density 0.64); and in T3, the number of non-zero edges were 43 (density 0.55). The NCT test showed that although the network densities varied between T1 and T3, the differences in overall global strength were not statistically different between T1 and T2 (T1; 3.240 and T2; 4.311, [S] = 1.070, *P* = .098) or between T2 and T3 (T2; 4.311 and T3; 2.872, [S] = 1.439, *P* = .090). However, the NCT test showed that the network invariance that is, structure of the networks was statistically different between T1 and T2 ([M] = 0.218, *P* = .001) and between T2 and T3 ([M] = 0.192, *P* = .002).

**Figure 2.**
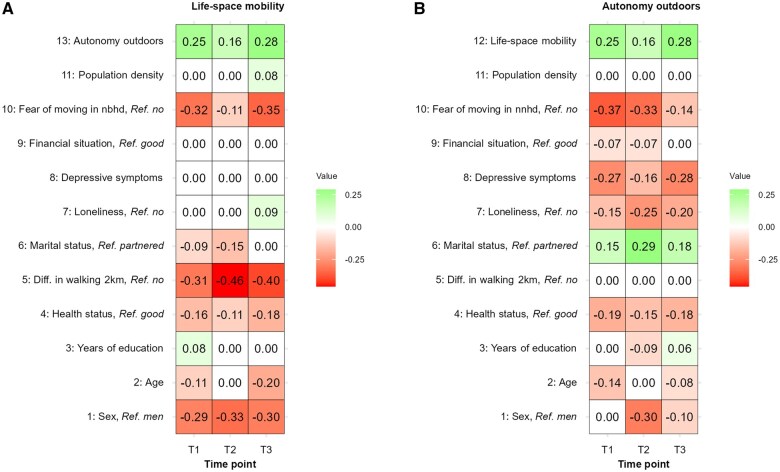
Relative importance of mobility determinants associated with (A) life-space mobility and (B) autonomy outdoors at different time points. The numbers in the boxes are the pairwise standardized absolute edge weights. A higher absolute number indicates a stronger association, while zero indicates no direct association. For ease of interpretation, the subscale of autonomy outdoors is inverted, that is, higher scores indicate greater autonomy. T1 = 2017–2018; T2 = 2020; T3 = 2021–2021.

Visual inspection of the networks revealed that life-space mobility and autonomy outdoors were influenced by partially distinct determinants. A heat map of standardized absolute pairwise edge weights was generated to facilitate visual inspection (Figure 2). A consistent positive association between life-space mobility and autonomy outdoors was observed at different time-points. Regarding life-space mobility (Figure 2A), difficulty walking 2 km and being a woman were strongly associated with lower life-space mobility throughout T1–T3. Fear of moving in the neighborhood also showed a consistent negative association with life-space mobility, with some weakening at the onset of the pandemic at T2. Older age showed a negative association with life-space mobility at T1 and T3, but not at T2, while not having a partner showed a negative association with life-space mobility at T1 and T2. However, according to Benjamini–Hochberg corrected tests for individual edge comparisons between networks of the NCT, no statistically significant differences in individual edges between the networks were observed (Supplementary Table S2)

In terms of autonomy outdoors (Figure 2B), being a woman was most strongly associated with lower autonomy outdoors at the onset of the pandemic in T2, while older age showed a negative association with autonomy outdoors at T1 and T3, but not at T2. Poorer self-rated health, more depressive symptoms, feeling lonely, and fear of moving around the neighborhood showed consistent associations with lower autonomy outdoors, with some variation over time. Not having a partner was positively associated with autonomy outdoors throughout T1–T3. According to Benjamini–Hochberg corrected tests for individual edge comparisons between networks, statistically significant (*P* < .05) differences between networks T1 and T2 were observed only for the edges between sex, age, and health status with autonomy outdoors. From T2 to T3, no statistically significant differences in individual edges between the networks were observed (Supplementary Table S2).

According to the bootstrap analysis, the estimated strengths and bootstrapped means were similar for most edges. The strongest observed edges with life-space mobility and autonomy outdoors showed good stability at different time points. However, some CIs for other edge weights in the networks are quite wide, which means that the estimation of edge weights and their rank order should be interpreted with caution (Supplementary Figures S1–S3, see online supplementary material for a color version of this figure). Bootstrapped differences between edge weights that were not zero at different time points are shown in the supplementary material (Figures S4–S6, see online supplementary material for a color version of this figure). In addition, sensitivity analyses were performed to compare imputed results with complete cases, with no significant differences observed (data not shown).

## Discussion

At the onset of the pandemic in 2020, both life-space mobility and autonomy outdoors declined, but only autonomy outdoors showed a statistically significant recovery approximately two years later. Our analyses revealed that the COVID-19 pandemic significantly influenced the overall network structures of out-of-home mobility and its underlying determinants over time, highlighting changes and dynamic interactions between them across different contexts. However, despite changes in the overall network structures between the time points, the determinants directly associated with life-space mobility and autonomy outdoors varied only slightly, with differences observed particularly for autonomy outdoors. During the onset of the COVID-19 pandemic, the relative importance of sex for autonomy outdoors became pronounced. In contrast, the importance of age and self-rated health decreased. Additionally, psychosocial determinants showed consistent associations with autonomy outdoors throughout the study period. Regarding life-space mobility, difficulty in walking and sex were its strongest determinants throughout the study period, with no statistically significant differences observed in individual edges between the networks.

The positive association between life-space mobility and autonomy outdoors observed throughout the study, along with findings suggesting that they are influenced by partly different underlying factors, supports the view that they represent related but distinct dimensions of out-of-home mobility. Our previous research showed that perceived opportunities for out-of-home mobility, that is, autonomy outdoors, declined more uniformly across individuals, regardless of factors such as lower extremity performance or driving status. In contrast, these factors appeared to buffer the decline in actual implementation of those opportunities, as reflected in life-space mobility.^13^ This distinction may also explain why a statistically significant recovery was observed only in perceived autonomy outdoors after the easing of the initial strict COVID-19 restrictions: perceptions may respond more directly to changes in policy and environmental support, whereas actual life-space mobility remains more closely tied to individual capacity and personal resources.

Due to the limited sample size, stratified network analysis by sex was not feasible. However, repeated measures ANOVA revealed that women consistently reported lower levels of autonomy outdoors across all time points, with a more pronounced decline at the onset of the pandemic in 2020. This pattern was also evident in the network analysis, in which sex became a stronger determinant of autonomy outdoors during the early phase of the pandemic. Several factors may explain the observed differences, especially at the onset of the pandemic. A previous cross-national study in eight countries found that women were more likely to perceive COVID-19 as a serious health threat, support restrictions, and comply with them.^45^ The increased concern and adherence to restrictions may have contributed to reduced perceived autonomy outdoors among older women. Compared to men, women are also more likely to rely on public transportation and drive less frequently,^46^ which potentially reduced their adaptability in maintaining out-of-home mobility during the pandemic. The closure of destinations and suspension of social activities further reduced the incentives to leave the home, thereby weakening environmental support for out-of-home mobility. This may have been more severe for women, reflecting the heterogeneous needs, preferences, and inequalities in social activities among older women and men.^47^ Conversely, the weakening association of age and self-rated health with autonomy outdoors at the onset of the pandemic may reflect the influence of COVID-19 restrictions and age-based guidelines. Treating all individuals aged 70 years and over as a homogeneous high-risk group may have influenced their perceived autonomy outdoors, regardless of individual differences in their actual age or health.

Loneliness and depressive symptoms consistently showed a negative association with perceived autonomy outdoors at different time points. Previous studies have indicated that individuals who feel lonely also report lower perceived autonomy outdoors, and that perceived environmental barriers may increase loneliness either directly or indirectly by reducing autonomy outdoors.^48^ Similarly, autonomy outdoors may mediate the relationship between depressive symptoms and reduced life-space mobility,^49^ which we also observed in our network models. Interestingly, not having a partner showed consistent positive association with autonomy outdoors, and a strong association with being a woman. People who live alone may have more freedom to choose their destinations and activities at any given time, and especially for older women, living alone does not necessarily pose a similar health threat as it does for men.^50^ Additionally, factors such as different attitudes toward the COVID-19 pandemic or concerns about a spouse’s health may have influenced perceived autonomy outdoors more for those in relationships than for those living alone. However, these explanations are speculative and warrant further investigation.

In the network analyses, difficulty in walking and sex were the most strongly associated with life-space mobility across all time points. These findings align with pre-pandemic research,^9^^,^^10^^,^^51^ which identified lower extremity performance and sex as strong determinants of life-space mobility. Repeated measures ANOVA further supported the role of sex, showing that women consistently reported lower levels of life-space mobility throughout the study period and experienced a greater decline at the onset of the pandemic compared to men. However, women also demonstrated a greater recovery in life-space mobility in 2021–2022. Several factors may contribute to these observed sex differences. Women tend to have lower physical functioning compared to men, which may contribute to difficulties in physical function and restricted life-space mobility.^51^ Additionally, sociocultural norms and historical gender roles may play a role as many older women grew up in contexts where women’s role in domestic tasks were emphasized, and engagement in independent travel, particularly driving, was less common.^52^^,^^53^ Regarding the greater decline observed at the onset of the pandemic, our previous research suggests that better lower extremity performance, along with driving, helped older adults to maintain greater life-space mobility during the pandemic. Notably, women represented the majority of non-drivers in our sample,^13^ which likely limited their opportunities for independent out-of-home mobility. This disadvantage may have been exacerbated by a more cautious attitude toward the pandemic, as discussed earlier.^45^ In terms of recovery, adaptation to living with the pandemic and the easing of restrictions in 2021–2022 may have had a greater positive impact on women’s life-space mobility, as men’s life-space mobility had already undergone a more modest change.

Previous literature on systemic resilience suggests that in a network of interdependent elements, increasing correlations between different elements over time may indicate systemic vulnerability and risk of systemic collapse.^54^ In the context of aging research, our group has previously shown that increasing density of the intrinsic capacity networks in older adults and those with lower self-rated health may indicate a loss of systemic resilience with aging and declining health.^20^ Similarly, Hao et al. showed that frail individuals had increased correlations and connections within frailty biomarker networks compared to robust and pre-frail groups, suggesting increased dependencies between the physiological systems underlying frailty.^18^ In this study, network density appeared to increase particularly from baseline to the onset of the pandemic, although no statistically significant difference was observed. It can be speculated that when external support for out-of-home mobility is disrupted, the different determinants of mobility may become increasingly interdependent, making dysfunction in one determinant more likely to affect others and ultimately impact overall mobility. However, this hypothesis needs further exploration in future studies.

A strength of this study is the use of network models to capture the complexity of older adults’ out-of-home mobility and its underlying determinants. This approach allowed us to examine the complex interactions among multiple mobility determinants related to two dimensions of out-of-home mobility—life-space mobility and autonomy outdoors—while also accounting for temporal changes in associations related to the COVID-19 pandemic. Another strength is the population-based sample of relatively old, community-dwelling older adults and data from three time points, with timely data collection related to different phases of the COVID-19 pandemic. This allowed us to examine the impact of different phases of the pandemic on the out-of-home mobility of older adults. Despite these strengths, several limitations should be considered. Due to the constraints imposed by the COVID-19 pandemic in 2020, only self-reported variables collected via postal questionnaires could be used in the second data collection in 2020. This limited the available variables and constrained the selection to those ensuring comparability of the networks across time points. Consequently, for example, cognition had to be excluded from the networks, and other variables, like physical determinants, relied on self-reports. Additionally, the preprocessing of categorical variables into binary form may have resulted in some further loss of information, potentially compromising their ability to accurately capture the intended constructs. Also, the relatively small sample size prevented stratified analysis, for example, by sex. Previous non-response analyses of the AGNES study have shown that participants in 2020 and 2021–2022 were generally younger and healthier,^13^^,^^23^ which may affect the generalizability of the results to older individuals with poorer health and physical function. In the future, it will be important to replicate these findings with larger study samples and to include more performance-based variables in the analyses.

## Conclusions

Life-space mobility and autonomy outdoors represent related yet distinct dimensions of out-of-home mobility among older adults, each influenced in part by different underlying determinants. Perceived possibilities for out-of-home mobility may respond more directly to changes in external conditions, whereas the actual implementation of out-of-home mobility depends more on individual capacities and personal resources. This study highlights the importance of investigating the temporal interactions between mobility determinants and out-of-home mobility, as changes in external conditions can alter these associations. The findings also suggest that women may be particularly vulnerable to declines in both perceived and actual out-of-home mobility when prevailing conditions that support out-of-home mobility suddenly change. This highlights the need for targeted strategies to support the out-of-home mobility of older adults, especially those with more limited resources, both in unexpected situations and in long-term planning for age-friendly environments.

## Supplementary Material

glaf162_Supplementary_Data
