# Supplementary material for: Determinants of mobility in community-dwelling older adults: a network analysis before, during, and two years after the onset of the COVID-19 pandemic
Source: J Gerontol A Biol Sci Med Sci. 2025 Jul 22;80(8):glaf162. doi: 10.1093/gerona/glaf162 (PMC12360778; doi:10.1093/gerona/glaf162)
Supplement: glaf162_Supplementary_Data [file glaf162_supplementary_data.docx]

**Supplementary Material**

Descriptive information

**Supplementary Table 1.** Coding and reference categories for binary variables used in analyses

NetworkComparisonTest

**Supplementary Table 2.** Benjamini-Hochberg corrected tests for the comparison of individual edges between networks of the NetworkComparisonTest

Non-parametric bootstrap stability of edge weight estimates

**Supplementary Figure 1.** Stability of the edge weight parameters and associated bootstrapped 95% confidence intervals (CIs) for the network at T1 2017–2018. The red line represents the true sample values, and the black line represents the bootstrapped means. The edges are ordered from the connection with the least weight to the one with the greatest weight on the X axis. The grey area represents the bootstrapped 95% CIs of the edge weights.

**Supplementary Figure 2.** Stability of the edge weight parameters and associated bootstrapped 95% confidence intervals (CIs) for the network at T2 2020. The red line represents the true sample values, and the black line represents the bootstrapped means. The edges are ordered from the connection with the least weight to the one with the greatest weight on the X axis. The grey area represents the bootstrapped 95% CIs of the edge weights.

**Supplementary Figure 3.** Stability of the edge weight parameters and associated bootstrapped 95% confidence intervals (CIs) for the network at T3 2021–2022. The red line represents the true sample values, and the black line represents the bootstrapped means. The edges are ordered from the connection with the least weight to the one with the greatest weight on the X axis. The grey area represents the bootstrapped 95% CIs of the edge weights.

Non-parametric bootstrapped difference test between edges

**Supplementary Figure 4.** Results of the bootstrapped difference test (p < 0.05) between edge weights that were non-zero in the estimated network at T1 2017–2018. Grey boxes correspond to edges that are not statistically different from each other. Black boxes correspond to edges that are statistically different from each other.

**Supplementary Figure 5.** Results of the bootstrapped difference test (p < 0.05) between edge weights that were non-zero in the estimated network at T2 2020. Grey boxes correspond to edges that are not statistically different from each other. Black boxes correspond to edges that are statistically different from each other.

**Supplementary Figure 6.** Results of the bootstrapped difference test (p < 0.05) between edge weights that were non-zero in the estimated network at T3 2021–2022. Grey boxes correspond to edges that are not statistically different from each other. Black boxes correspond to edges that are statistically different from each other.

S**upplementary Table 1.** Coding and reference categories for binary variables used in analyses

| **Variable** | **Coding and reference category** | |
| --- | --- | --- |
| Sex | **0** Man (ref.) | **1** Woman |
| Health status | **0** Good (ref.) | **1** Fair or poor |
| Difficulties walking 2 km | **0** No difficulties (ref.) | **1** At least some difficulties |
| Marital status | **0** Partnered (ref.) | **1** Not partnered |
| Loneliness | **0** Never or very rarely (ref.) | **1** At least sometimes |
| Financial situation | **0** Good (ref.) | **1** Moderate or poor |
| Fear of moving in the neighbourhood | **0** No (ref.) | **1** Yes |

**Supplementary Table 2.** Benjamini-Hochberg corrected tests for the comparison of individual edges between networks of the NetworkComparisonTest

|  | **Life-space**  **mobility** | | **Autonomy**  **outdoors** | |
| --- | --- | --- | --- | --- |
|  | **T1 vs. T2** | **T2 vs. T3** | **T1 vs. T2** | **T2 vs. T3** |
|  | p-value | p-value | p-value | p-value |
| Life-space mobility / Autonomy outdoors | .351 | .164 | .351 | .164 |
| **Sociodemographic** |  |  |  |  |
| Sex | .844 | .454 | **.026** | .164 |
| Age | .607 | .111 | **.026** | .078 |
| Total years of education | 1.00 | 1.00 | .156 | .078 |
| **Physical determinants** |  |  |  |  |
| Health status | 1.00 | 1.00 | **.039** | .078 |
| Difficulties in walking 2 km | .746 | 1.00 | .156 | .094 |
| **Psychosocial determinants** |  |  |  |  |
| Marital status | .623 | .301 | .228 | .382 |
| Loneliness | .748 | .164 | 1.00 | 1.00 |
| Depressive symptoms | 1.00 | 1.00 | .607 | .473 |
| **Financial determinants** |  |  |  |  |
| Financial situation | .239 | .444 | .227 | 1.00 |
| **Environmental determinants** |  |  |  |  |
| Fear in neighborhood | 1.00 | 1.00 | 1.00 | .912 |
| Population density | 1.00 | 1.00 | 1.00 | 1.00 |

Note: T1 = baseline 2017–2018, T2 = the onset of the COVID-19 pandemic in 2020, T3 = follow-up 2021–2022.

**
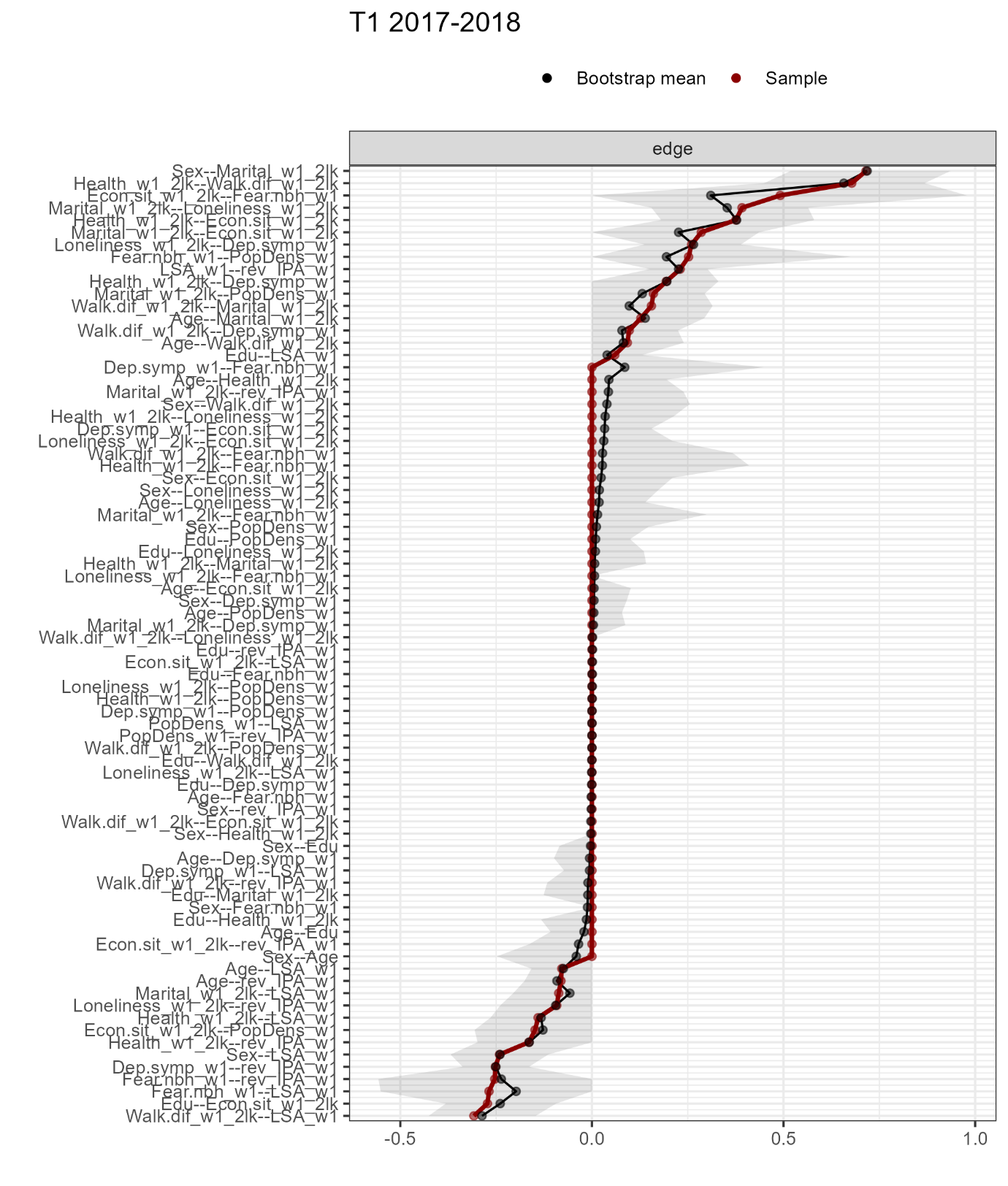
**

**Supplementary Figure 1.** Stability of the edge weight parameters and associated bootstrapped 95% confidence intervals (CIs) for the network at T1 2017–2018. The red line represents the true sample values, and the black line represents the bootstrapped means. The edges are ordered from the connection with the least weight to the one with the greatest weight on the X axis. The grey area represents the bootstrapped 95% CIs of the edge weights.

**
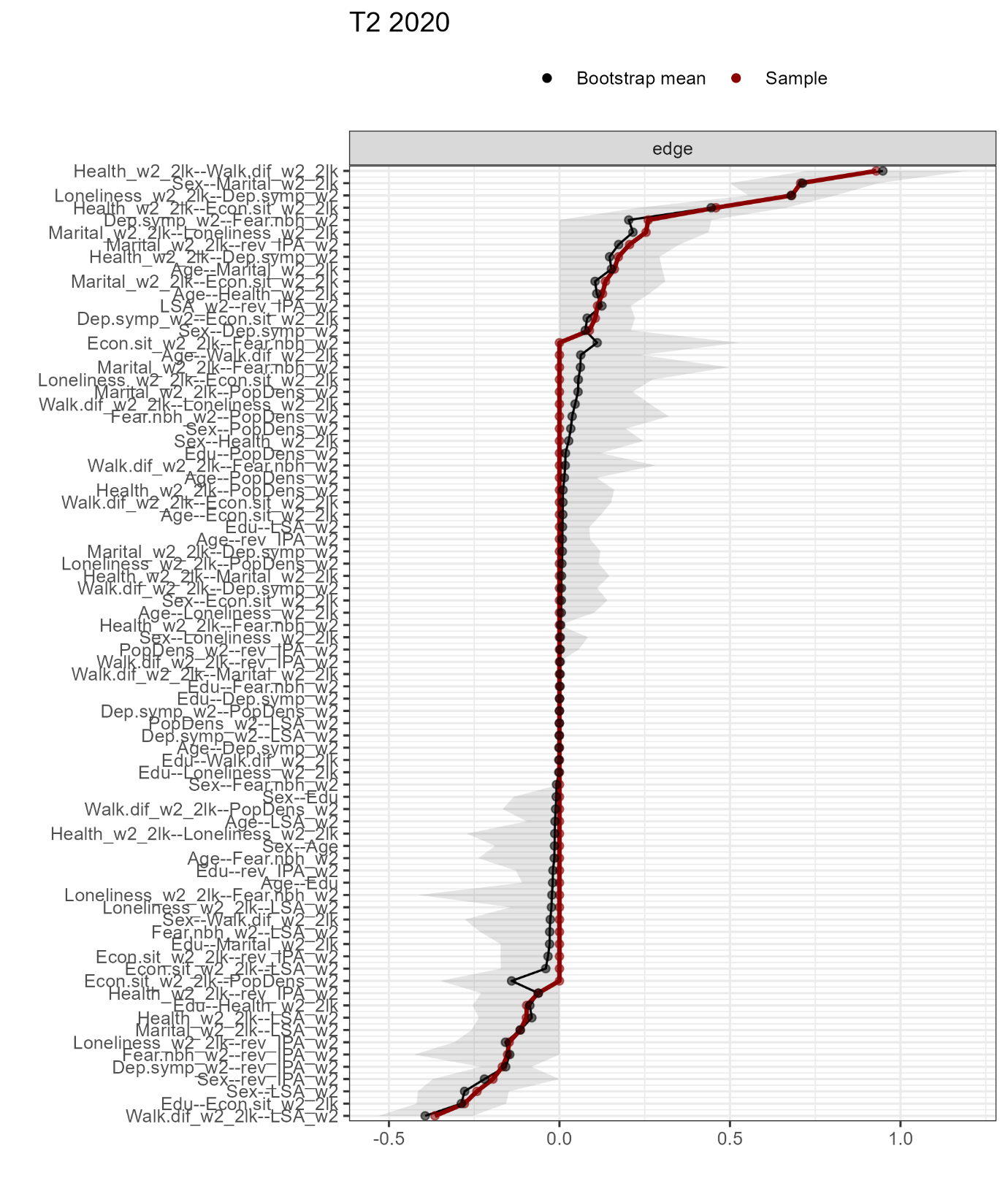
**

**Supplementary Figure 2.** Stability of the edge weight parameters and associated bootstrapped 95% confidence intervals (CIs) for the network at T2 2020. The red line represents the true sample values, and the black line represents the bootstrapped means. The edges are ordered from the connection with the least weight to the one with the greatest weight on the X axis. The grey area represents the bootstrapped 95% CIs of the edge weights.


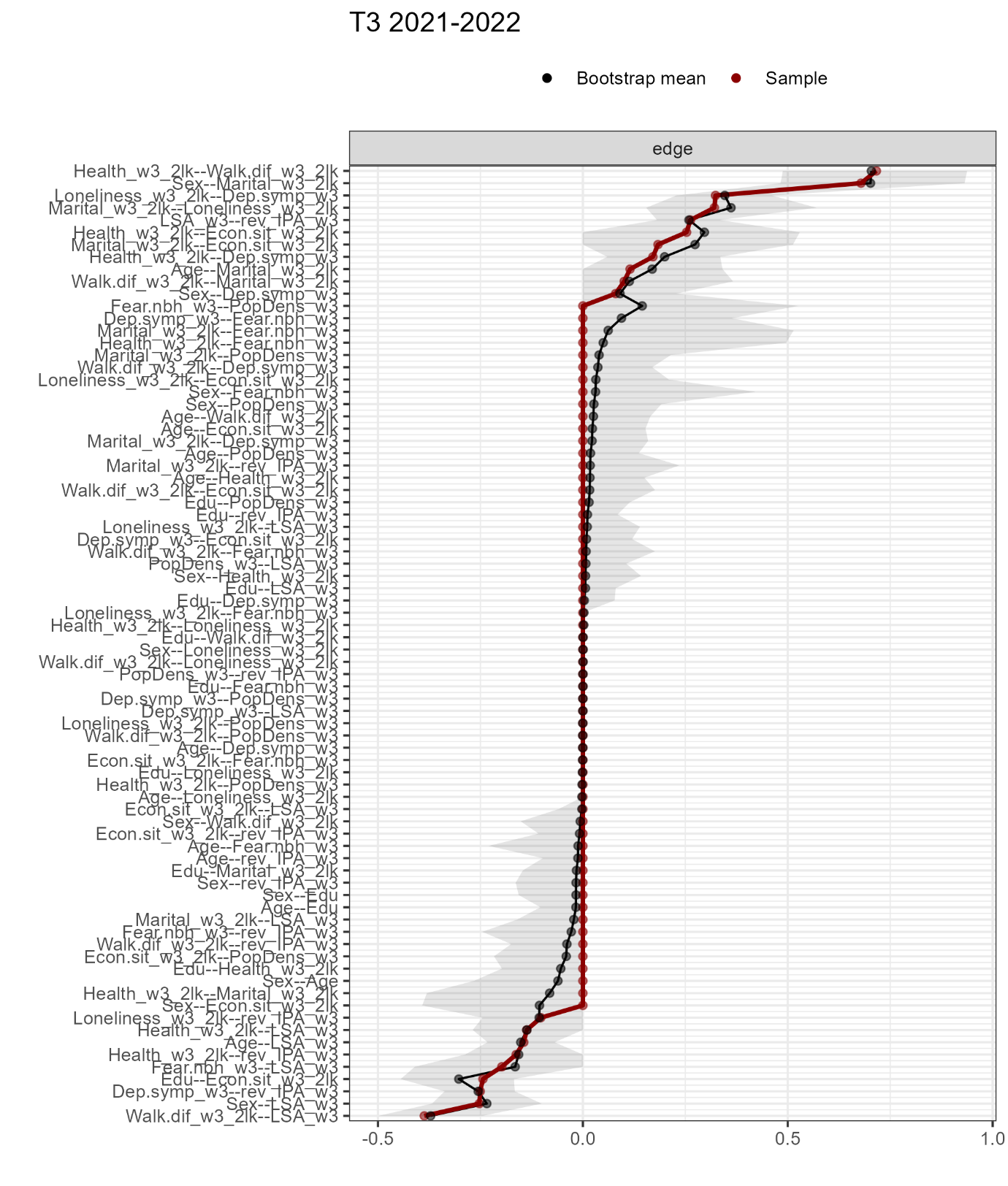


**Supplementary Figure 3.** Stability of the edge weight parameters and associated bootstrapped 95% confidence intervals (CIs) for the network at T3 2021–2022. The red line represents the true sample values, and the black line represents the bootstrapped means. The edges are ordered from the connection with the least weight to the one with the greatest weight on the X axis. The grey area represents the bootstrapped 95% CIs of the edge weights.


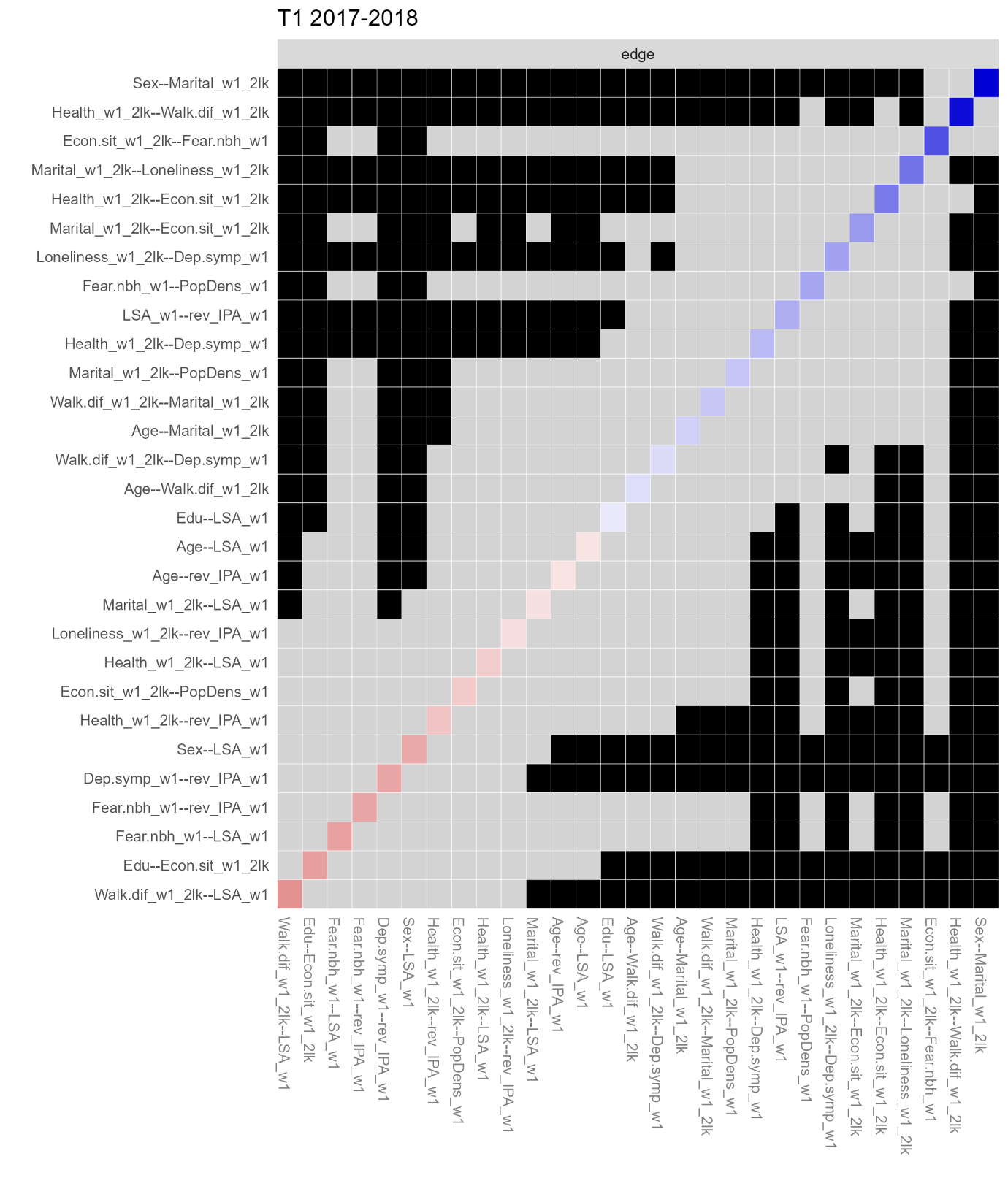


**Supplementary Figure 4.** Results of the bootstrapped difference test (p < 0.05) between edge weights that were non-zero in the estimated network at T1 2017–2018. Grey boxes correspond to edges that are not statistically different from each other. Black boxes correspond to edges that are statistically different from each other.


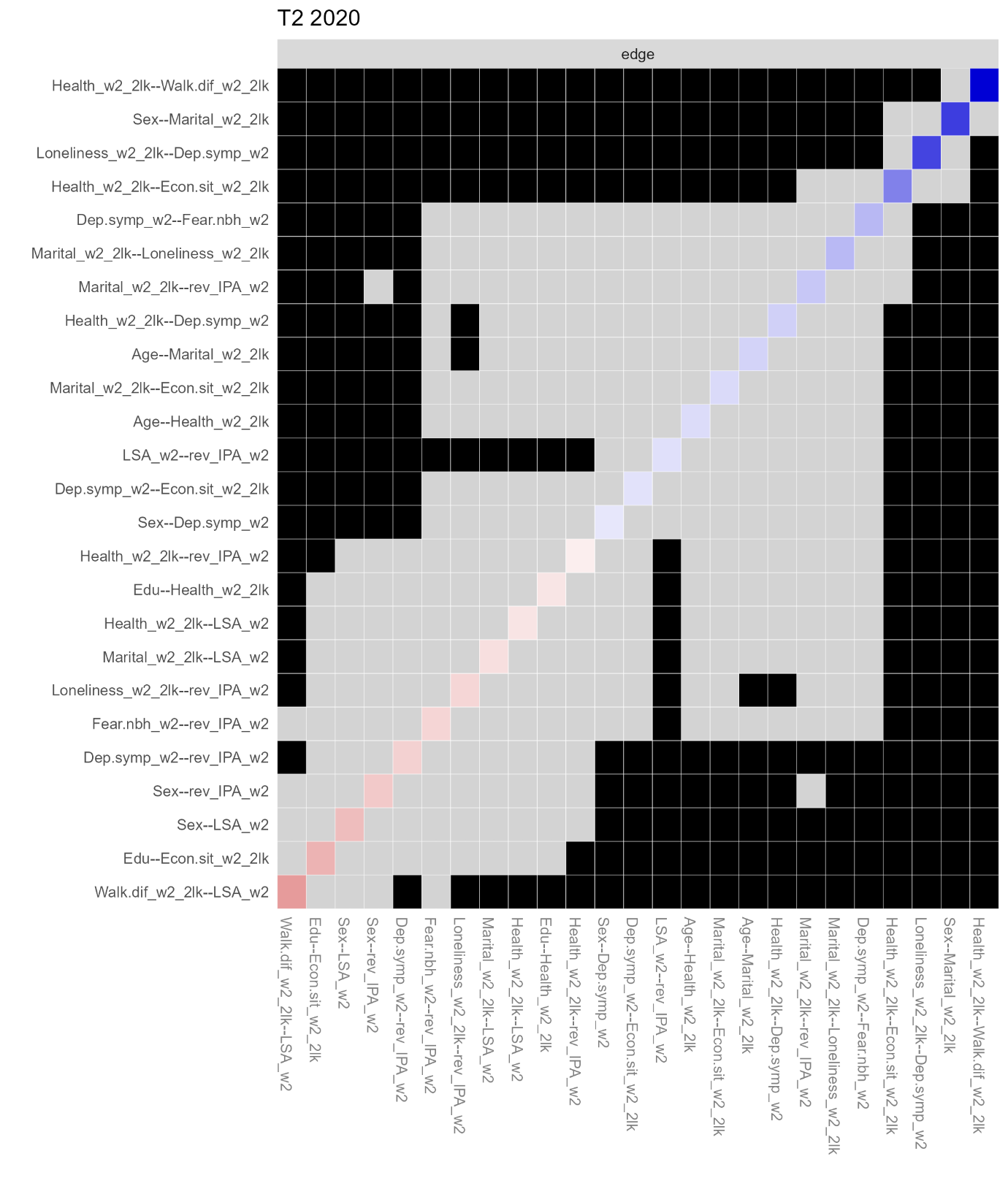


**Supplementary Figure 5.** Results of the bootstrapped difference test (p < 0.05) between edge weights that were non-zero in the estimated network at T2 2020. Grey boxes correspond to edges that are not statistically different from each other. Black boxes correspond to edges that are statistically different from each other.


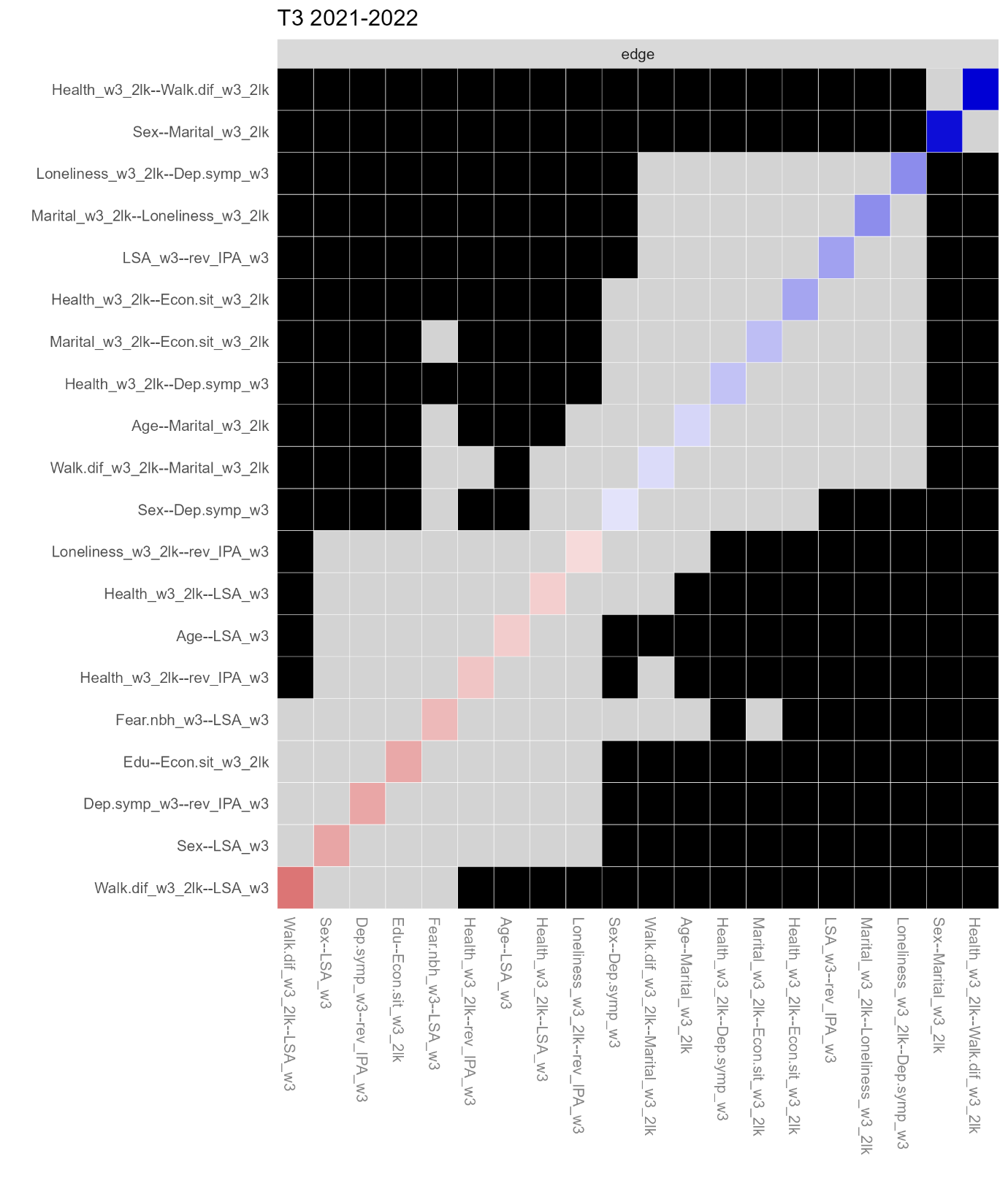


**Supplementary Figure 6.** Results of the bootstrapped difference test (p < 0.05) between edge weights that were non-zero in the estimated network at T3 2021–2022. Grey boxes correspond to edges that are not statistically different from each other. Black boxes correspond to edges that are statistically different from each other.
